# Bioinformatics and biomedical informatics with ChatGPT: year one review

**Published:** 2024-03-22

**Authors:** Jinge Wang, Zien Cheng, Qiuming Yao, Li Liu, Dong Xu, Gangqing Hu

**Affiliations:** 1 Department of Microbiology, Immunology & Cell Biology, West Virginia University, Morgantown, WV 26506, USA; 2 School of Computing, University of Nebraska-Lincoln, Lincoln, NE 68588, USA; 3 College of Health Solutions, Arizona State University, Phoenix, AZ 85004, USA; 4 Biodesign Institute, Arizona State University, Tempe, AZ 85281, USA; 5 Department of Electrical Engineer and Computer Science, Christopher S. Bond Life Sciences Center, University of Missouri, Columbia, MO 65211, USA

**Keywords:** ChatGPT, Bioinformatics, Biomedical Informatics

## Abstract

The year 2023 marked a significant surge in the exploration of applying large language model (LLM) chatbots, notably ChatGPT, across various disciplines. We surveyed the applications of ChatGPT in various sectors of bioinformatics and biomedical informatics throughout the year, covering omics, genetics, biomedical text mining, drug discovery, biomedical image understanding, bioinformatics programming, and bioinformatics education. Our survey delineates the current strengths and limitations of this chatbot in bioinformatics and offers insights into potential avenues for future development.

## 1. INTRODUCTION

In recent years, artificial intelligence (AI) has attracted tremendous interest across various disciplines, emerging as an innovative approach to tackling scientific challenges[1]. The surge in data generated from both public and private sectors, combined with the rapid advancement in AI technologies, has facilitated the development of innovative AI-based solutions and accelerated scientific discoveries[1–3]. The launch of the Chat Generative Pre-trained Transformer (ChatGPT) to the public towards the end of 2022 marked a new era in AI. The biomedical research community embraces this new tool with immense enthusiasm. In 2023 alone, at least 2,074 manuscripts were indexed in PubMed when searching with the keyword “ChatGPT”. These studies demonstrate that ChatGPT and similar models have great potential to transform many aspects of education, biomedical research, and clinical practices[4–7].

The core of ChatGPT is a large-language model (LLM) trained on a vast corpus of text data from the internet, including biomedical literature and code[8]. Its ability to comprehend and respond in natural language positions ChatGPT as a valuable tool for biomedical text-based inquiry[9]. Particularly noteworthy is its potential in assisting bioinformatics analysis, enabling scientists to conduct data analyses via verbal instructions[10–12]. Surprisingly, a search on PubMed using the keywords “ChatGPT” and “bioinformatics” returned only 30 publications. While this number could have been underestimated by limiting the search to PubMed, and a few hundred related articles are probably archived as preprints or under review, it still suggests that the application of this innovative tool in bioinformatics is relatively underexplored compared to other areas of biomedical research.

**Figure 1:**
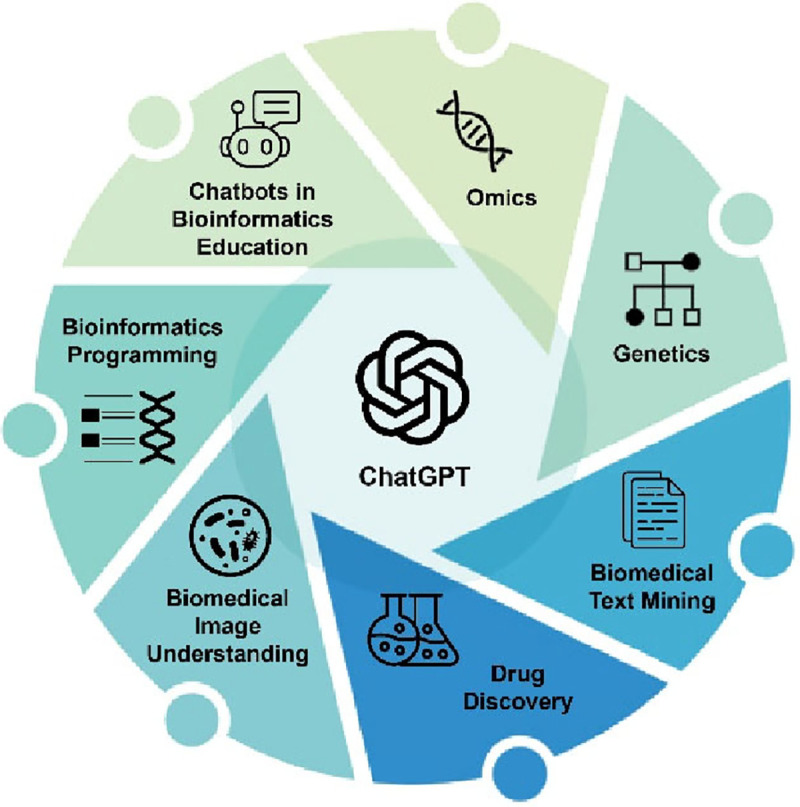
Areas within bioinformatics and biomedical informatics explored in this review.

In this review, we summarize recent advancements, predominantly within 2023, in the application of ChatGPT across a broad spectrum of bioinformatics and biomedical informatics topics, including omics, genetics, biomedical text mining, drug discovery, biomedical images, bioinformatics programming, and bioinformatics education (Figure 1). As the topics are relatively new, this survey included not only publications in journals but also preprints in various archive platforms. Our objective is to encapsulate recurring themes from independent works within the same topic or across multiple topics, pinpointing prospective avenues for further exploration. Additionally, this review allows us to identify challenges in integrating chatbots into bioinformatics, such as inefficiency in prompt generation, uncertainty in responses, and concerns over data privacy[13–15]. The insights from this analysis are anticipated to benefit other domains where the integration of chatbot technology is actively pursued.

## 2. OMICS

Omics techniques are extensively employed in biomedical research, generating vast amounts of data that necessitate careful analysis to uncover significant discoveries. A novel application of GPT models in transcriptomics has been to annotate cell types in single-cell RNA sequencing data[16], traditionally a labor-intensive and expertise-demanding task. Leveraging the wealth of online texts that offer detailed descriptions of signature genes for various cell types, ChatGPT can efficiently identify cell types based on a brief list of marker genes, as demonstrated by Hou and Ji [16]. When evaluated across datasets encompassing numerous tissues and cell types, ChatGPT demonstrates strong concordance with manual annotations and surpasses several conventional methods[16]. However, given the undisclosed nature of GPT’s training data and the potential for AI-generated errors, expert validation is recommended before leveraging its annotations in further research.

The task of identifying protein-coding regions within DNA sequences plays a crucial role in genome annotation[17]. A key step in this process involves the extraction of all open reading frames (ORFs), defined as continuous stretches of DNA that start with a start codon and end with a stop codon, without any intervening stop codons. In a test involving three partial sequences from the Vaccinia virus, ChatGPT-4 demonstrates its ability to identify potential ORFs[18]. However, accurately pinpointing the longest ORFs requires additional instructions based on the Chain-of-Thought (CoT) approach (See Table 1 for terminologies cited in this review). The capacity of ChatGPT-4 to precisely assess the coding potential of each ORF remains to be investigated. Nonetheless, the chatbot correctly emphasizes that an ORF’s length does not inherently indicate its coding potential and experimental validation is necessary to confirm this potential[18].

Evaluating GPT models in genomics necessitates benchmark datasets with established ground truths. GeneTuring[19] serves this role with 600 questions related to gene nomenclature, genomic locations, functional characterization, sequence alignment, etc. When tested on this dataset, GPT-3 excels in extracting gene names and identifying protein-coding genes, while ChatGPT (GPT-3.5) and New Bing show marked improvements. Nevertheless, all models face challenges with SNP and alignment questions[19]. This limitation is effectively addressed by GeneGPT[20], which utilizes Codex to consult the National Center for Biotechnology Information (NCBI) database.

Bioinfo-Bench[21], currently under development, aims to assess LLMs in bioinformatics through 150 multiple-choice questions covering various topics, such as sequence analysis and phylogenetics, and 20 questions on RNA sequence mutations. ChatGPT-3.5 outperforms other LLMs, such as Llama-7B and Galactica-30B. However, its role in generating distractor options (wrong answers) for these questions might artificially boost its performance, suggesting a need for cautious interpretation when using it to self-evaluate.

## 3. GENETICS

In North America, 34% of genetic counselors incorporate ChatGPT into their practice, especially in administrative tasks[22]. This integration marks a significant shift towards leveraging AI for genetic counseling and underscores the importance of evaluating its reliability. Doung and Solomon[23] analyzed ChatGPT’s performance on multiple-choice questions in human genetics sourced from Twitter. The chatbot achieves a 70% accuracy rate and excels in tasks requiring memorization over critical thinking. Further analysis by Alkuraya [24] revealed ChatGPT’s limitations in calculating recurrence risks for genetic diseases. A notable instance involving cystic fibrosis testing showcases the chatbot’s ability to derive correct equations but falter in computation, raising concerns over its potential to mislead even professionals[24]. This aspect of plausible responses is also identified as a significant risk by genetic counselors[22].

These observations have profound implications for the future education of geneticists. It indicates a shift from memorization tasks to a curriculum that emphasizes critical thinking in varied, patient-centered scenarios, scrutinizing AI-generated explanations rather than accepting them at face value[25]. Moreover, it stresses the importance of understanding AI tools’ operational mechanisms, limitations, and ethical considerations essential in genetics[23]. This shift aims to prepare geneticists better for AI use, ensuring they remain informed on the benefits and risks of technology.

## 4. BIOMEDICAL TEXT MINING

**Figure 2:**
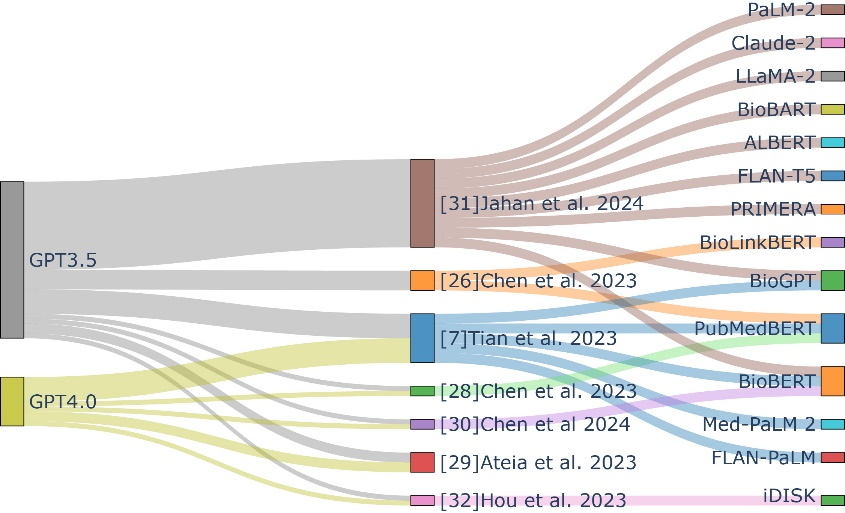
Overview of evaluation works on GPT models and comparison to state-of-the-art models in typical biomedical text mining tasks.

For biomedical text mining with ChatGPT, we first summarized works that evaluate the performance of ChatGPT in various biomedical text mining tasks and compared it to state-of-the-art (SOTA) models (Figure 2). Then, we explored how ChatGPT has been used to reconstruct biological pathways and prompting strategies used to improve performance.

### 4.1. PERFORMANCE ASSESSMENTS ACROSS TYPICAL TASKS

Biomedical text mining tasks typically include name entity recognition, relation extraction, sentence similarity, document classification, and question answering. Chen, Sun [26] assessed ChatGPT-3.5 across 13 publicly available benchmarks. While its performance in question answering closely matched SOTA models like PubmedBERT[27], ChatGPT-3.5 showed limitations in other tasks, with similar observations made for ChatGPT-4[7, 28, 29]. Extensions to sentence classification and reasoning revealed that ChatGPT was inferior to SOTA pretrained models like BioBERT[30]. These studies highlight the limitations of ChatGPT in some specific domains of biomedical text mining where domain-optimized language models excel. Nevertheless, when the training sets with task-specific annotations are not sufficient, zero-shot LLMs, including ChatGPT-3.5 outperform SOTA finetuned biomedical models[31].

Biomedical Knowledge Graphs (BKGs) have emerged as a novel paradigm for managing large-scale, heterogeneous biomedical knowledge from expert-curated sources. Hou, Yeung [32] evaluated ChatGPT’s capability on question and answering tasks using topics collected from the “Alternative Medicine” subcategory on “Yahoo! Answers” and compare to the Integrated Dietary Supplements Knowledge Base (iDISK)[33]. While ChatGPT-3.5 showed comparable performance to iDISK, ChatGPT-4 was superior to both. However, when tasked to predict drug or dietary supplement repositioned for Alzheimer’s Disease, ChatGPT primarily responded with candidates already in clinical trials or existing literature. Moreover, ChatGPT’s efforts to establish associations between Alzheimer’s Disease and hypothetical substances were less than impressive. This highlights ChatGPT’s limitations in performing novel discoveries or establishing new entity relationships within BKGs.

ChatGPT’s underperformance in some specific text mining tasks against SOTA models or BKGs identifies areas for enhancement; On the other hand, finetuning LLMs, although beneficial, remains out of reach for most users due to the high computational demand. Therefore, techniques like prompt engineering, including one/few-shot in-context learning and CoT, can be more practical to improve LLM efficiency in text mining tasks[26, 28, 30, 34]. For instance, incorporating examples with CoT reasoning enhances the performances over both zero-shot (no example) and plain examples in sentence classification and reasoning tasks[30] as well as knowledge graph reconstruction from literature titles[35]. However, simply increasing the number of examples does not always correlate with better performance[28, 30]. This underscores another challenge in optimizing LLMs for specialized text mining tasks, necessitating more efficient prompting strategies to ensure consistent reliability and stability.

### 4.2. BIOLOGICAL PATHWAY MINING

Another emerging application of biomedical text mining from LLMs is to build biological pathways. The ChatPathway project[36] assessed ChatGPT-3.5’s intrinsic capability to predict biochemical reactions and map out metabolic or regulatory pathways based on given reactants and enzymes. Using KEGG annotations as benchmarks, it reported modest prediction accuracies: 24.1% for biochemical reactions, 24.6% to 42.3% for metabolic pathways, and 20.1% to 39.9% for regulatory pathways. Azam, Chen [37] conducted a broader assessment of mining gene interactions and biological pathways across 21 LLMs, including seven Application Programming Interface (API)-based and 14 open-source models. ChatGPT-4 and Claude-Pro emerged as leaders, though they only achieved F1 scores less than 0.5 for gene relation predictions and a Jaccard index less than 0.3 for pathway predictions. Another evaluation work on retrieving protein-protein interaction (PPI) from sentences reported a modest F1 score for both GPT-3.5 and GPT-4 for base prompts[38]. All the studies underscore the inherent challenges generic LLMs face in delineating gene relationships and constructing complex biological pathways from biomedical text without prior knowledge or specific training.

Nevertheless, the capabilities of ChatGPT in knowledge extraction and summarization present promising avenues for database curation support. Tiwari, Matthews [39] explored its utility in the Reactome curation process, notably in identifying potential proteins for established pathways and generating comprehensive summaries. For the case study on the circadian clock pathway, ChatGPT proposed 13 new proteins, five of which were supported by the literature but overlooked in traditional manual curation. When summarizing pathway from multiple literature extracts, ChatGPT struggled to resolve contradictions, but gained improved performance when inputs contained in-text citations. Similarly, the use of ChatGPT for annotating long non-coding RNAs in the EVLncRNAs 3.0 database[40] faces issues with inaccurate citations. Both works demonstrate the challenge of direct use of ChatGPT in assisting in database curation.

Supplementing ChatGPT with domain knowledge or literature has been shown to mitigate some of its intrinsic limitations. The inclusion of a protein dictionary in prompts improves performance for GPT-3.5 and GPT-4 in PPI task[38]. Chen, Li [41] augmented ChatGPT with literature abstracts to identify genes involved in arthrofibrosis pathogenesis. Similarly, Fo, Chuah [42] supplied GPT-3.5 with plant biology abstracts to uncover over 400,000 functional relationships among genes and metabolites. This domain knowledge/literature-backed approach enhances the reliability of chatbots in text generation by reducing AI hallucination[43, 44].

Addressing LLMs’ intrinsic limitations can also involve sophisticated prompt engineering. Chen, Gao [45] introduced an iterative prompt optimization procedure to boost ChatGPT’s accuracy in predicting gene-gene interactions, utilizing KEGG pathway database as a benchmark. Initial tests without prompt enhancements showed a performance decline along with ChatGPT’s upgrades from March to July in 2023, but the strategic role and few-shot prompts significantly countered this trend. The iterative optimization process, which employed the tree-of-thought methodology[46], achieved notable improvements in precision and F1 scores. These experiments demonstrate the value of strategic prompt engineering in aligning LLM outputs with complex biological knowledge for better performance.

## 5. DRUG DISCOVERY

Drug discovery is a complex and failure-prone process that demands significant time, effort, and financial investment. The emerging interest in ChatGPT’s potential to facilitate drug discovery has captivated the pharmaceutical community[47–50]. Recent studies have showcased the chatbot’s proficiency in addressing tasks related to drug discovery. GPT-3.5, for example, has been noted for its respectable accuracy in identifying associations between drugs and diseases[51]. Furthermore, GPT models exhibit strong performance in tasks related to textual chemistry, such as generating molecular captions, but face challenges in tasks that require accurate interpretation of the Simplified Molecular-Input Line-Entry System (SMILES) strings[52]. Research by Juhi, Pipil [53] highlighted ChatGPT’s partial success in predicting and elucidating drug-drug interactions (DDIs). When benchmarked against two clinical tools, GPT models achieved an accuracy rate of 50–60% in DDI prediction, showing a 20–30% improvement with further optimization[54]. When evaluated using the DDI corpus[55], ChatGPT achieved an F1 score of 52%[26]. In more rigorous assessments, ChatGPT was unable to pass various pharmacist licensing examinations[56–58]. It also shows limitations in patient education and in recognizing adverse drug reactions[59]. These findings suggest that, although ChatGPT offers valuable support in drug discovery, its capacity to tackle complex challenges is ineffective and necessitates close human oversight.

**Figure 3:**
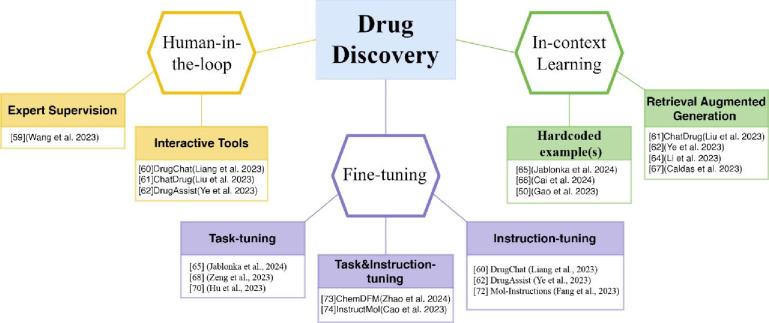
Key themes from the application of GPTs and other LLMs in drug discovery efforts.

In the following few sections, we will review three important aspects of LLM use in drug discovery (Figure 3). review examples and tools that facilitate a human-in-the-loop approach for reliable use of ChatGPT in drug discovery. Then we highlight the advances brought by strategic prompting using in-context learning with examples to increase response accuracy. Lastly, we summarize the progress of using task- and or instruction finetune to adapt a foundational model to specific tasks.

### 5.1. HUMAN-IN-THE-LOOP

The application of AI in drug development necessitates substantial expertise from human specialists for result refinement. This collaborative approach is illustrated in a case study focusing on the development of anti-cocaine addiction drugs aided by ChatGPT[60]. Throughout this process, GPT-4 assumes three critical roles in sparking new ideas, clarifying methodologies, and providing coding assistance. To enhance its performance, the chatbot is equipped with various plugins at each phase to ensure deeper understanding of context, access to the latest information, improved coding capabilities, and more precise prompt generation. The responses generated by the chatbot are critically evaluated with existing literature and expert domain knowledge. Feedback derived from this evaluation is then provided to the chatbot for further improvement. This iterative, human-in-the-loop methodology led to the identification of 15 promising multi-target leads for anti-cocaine addiction[60]. This example underscores the synergistic potential of human expertise and AI in advancing drug discovery efforts.

Several tools leveraging LLMs offer interactive interfaces to enhance molecule description and optimization. DrugChat[61] is a system akin to ChatGPT, offering interactive question-and-answer and textual explanations starting from drug graph representations. ChatDrug[62], on the other hand, is a framework built to streamline the process of drug editing through GPT APIs. It features a prompt design module equipped with a collection of template prompts. Furthermore, it incorporates a retrieval and domain feedback module, which pulls examples from external databases to ensure that the response is grounded in real-world examples. Additionally, ChatDrug includes a conversational module dedicated to interactive refinement. This module allows for the integration of feedback from domain experts through iteration, ensuring that each suggestion is safeguarded through expert scrutiny. DrugAssist[63] adapts a similar approach for molecule optimization by utilizing external database retrieval for hints and allowing iterative refinement with expert feedback. The process of iterative refinement, supported by example retrieval from external databases as contextual hints, enhances the model’s accuracy and relevance to practical applications.

### 5.2. IN-CONTEXT LEARNING

In-context learning (ICL) enhances chatbots’ responses by leveraging examples from a domain knowledgebase through prompting without finetuning a foundation model[64]. This approach utilizes examples closely aligned with the subject matter to ground the responses of LLMs with relevant domain knowledge[62, 63, 65]. Evaluating LLMs’ capabilities across various chemistry-related tasks has shown that including contextually similar examples results in superior outcomes compared to approaches that use no example or employ random sampling; The performance of these models improves progressively with the inclusion of additional examples[52, 65, 66]. ICL also boosts the accuracy in more complex regression tasks, rendering GPT-4 competitively effective compared to dedicated machine learning models[67, 68]. Lastly, instead of specific examples, enriching the context with related information—such as disease backgrounds and synonyms in a fact check task on drug-disease associations[51] —also augments response accuracy. These examples, with in-context learning and context enrichment, underscore the critical role of domain-knowledge in improving the quality and reliability of LLM responses in drug discovery tasks.

### 5.3. INSTRUCTION FINETUNING

Task-tuning language models for specific tasks within drug discovery has shown considerable promise, as evidenced by several recent projects. ChatMol[69] is a chatbot based on the T5 model[70], finetuned with experimental property data and molecular spatial knowledge to improve its capabilities in describing and editing target molecules. Task-tuning GPT-3 has demonstrated notable advantages over traditional machine learning approaches, particularly in tasks where training data is small[66]. Task-tuning also significantly improves GPT-3 in extracting DDI triplets, showcasing a substantial F1 score enhancement over GPT-4 with few-shots[71]. These projects demonstrate that task-tuning of foundation models can effectively capture the complex knowledge at the molecule level relevant to drug discovery.

Instruction tuning diverges from task tuning by training an LLM across a spectrum of tasks using instruction-output pairs and enables the model to address new, unseen tasks[72]. DrugAssist[63], a Llama-2–7B-based model, though instruction-tuned with data with individual molecule properties, achieved competitive results when simultaneously optimizing multiple properties. Similarly, DrugChat[61], a Vicuna-13b-based model instruction-tuned with examples from databases like ChEMBL and PubChem, effectively answered open-ended questions about graph-represented drug compounds. Mol-Instructions[73], a large-scale instruction dataset tailored for the biomolecular domain, demonstrated its effectiveness in finetuning models like Llama-7B on a variety of tasks, including molecular property prediction and biomedical text mining.

Task-tuning may be combined with instruction tuning to synergize the strength of each. ChemDFM[74], pre-trained on LLaMa-13B with a chemically rich corpus and further enhanced through instruction tuning, exceled in a range of chemical tasks, particularly in molecular property prediction and reaction prediction, outperforming models like GPT-4 with in-context learning. InstructMol[75] is a multi-modality instruction-tuning-based LLM. It has a two-stage tuning process, first by instruction tuning with molecule graph-text caption pairs to integrate molecule knowledge and then by task-specific tuning for three drug discovery-related molecular tasks. Applied to Vicuna-7B, InstructMol surpassed other leading open-source LLMs and narrows the performance gap with specialized models[75]. These developments underscore the effectiveness of both task and instruction tuning as strategies for enhancing generalized foundation models with domain-specific knowledge to address specific challenges in drug discovery.

## 6. BIOMEDICAL IMAGE UNDERSTANDING

In recent advancements, multimodal AI models have garnered significant attention in biomedical research[76]. Released in late September 2023, GPT-4V(ision) has been the subject of numerous studies that explored its application in image-related tasks across various biomedical topics[77–83]. For biomedical images, GPT-4V exhibits a performance rivaling professionals in Medical Visual Question Answering[81, 82] and exceeds traditional image models in biomedical image classification[84]. For scientific figures, GPT-4V can proficiently explain various plot types and apply domain knowledge to enrich interpretations[85].

Despite the impressive performance, current evaluations reveal significant limitations. OpenAI acknowledges the limitation of GPT-4V in differentiating closely located text and making factual errors in an authoritative tone[86]. The model is not competent in perceiving visual patterns’ colors, quantities, and spatial relationships in scientific figures[85]. Image interpretation with domain knowledge from GPT-4V may risk “confirmation bias”[87]: either the observation or conclusion is incorrect, but the supporting knowledge is valid[85], or the observation or conclusion is correct, but the supporting knowledge is invalid/irrelevant[88]. Such biases are particularly concerning as users without requisite expertise might be easily misled by these plausible responses.

Prompt engineering has been instrumental in enhancing AI responses to text inputs. The emergence of GPT-4V emphasizes the need to develop equivalent methodologies for visual inputs to refine chatbots’ comprehension across modalities. The field of computer vision has already witnessed some progress in this direction[89]. Yang, Li [90] proposes visual referring prompting (VRP) by setting visual pointer references through directly editing input images to augment textual prompts with visual cues. VRP has proven effective in preliminary case studies, leading to the creation of a benchmark like VRPTEST[91] to evaluate its efficacy. Yet, a thorough, quantitative assessment of VRP’s impact on GPT-4V’s understanding of biomedical images remains to be explored.

## 7. BIOINFORMATICS PROGRAMMING

ChatGPT enables scientists who may not possess advanced programming skills to perform bioinformatics analysis. Users can articulate data characteristics, analysis details, and objectives in natural language, prompting ChatGPT to respond with executable code. In this context, we define “prompt bioinformatics”: the use of natural language instructions (prompts) to guide chatbots for reliable and reproducible bioinformatics data analysis through code generation[13]. This concept differs from the development of bioinformatics chatbot before the GPT era, such as DrBioRight[92] and RiboChat[93]. In prompt bioinformatics, the code is generated on the fly by the chatbot in response to a data analysis description. In addition, the generated code inherently varies across different chat sessions even for the same instruction, asking for method developments to ensure result reproducibility. Lastly, the concept covers a broad range of bioinformatics topics, particularly those in applied bioinformatics, where data analysis methods are relatively mature.

Early case studies showcase ChatGPT’s versatility in addressing diverse bioinformatics coding tasks, from aligning sequencing reads to constructing evolutionary trees[10], and excelling in introductory course exercises[12]. ChatGPT excels at writing short scripts that call existing functions with specific instructions[94]. However, it shows limitations in writing longer, workable code for more complex data analysis with errors often requiring domain-specific knowledge to spot for correction[94].

### 7.1. APPLICATION IN APPLIED BIOINFORMATICS

In applied bioinformatics, established methods for data analysis are prevalent used, enhancing the likelihood of their incorporation into LLM training datasets. Thus, applied bioinformatics emerges as a fertile ground for practicing prompt bioinformatics and evaluating its effectiveness. AutoBA[95], a Python package powered by LLMs, streamlined applied bioinformatics for multi-omics data analysis by autonomously designing analysis plans, generating code, managing package installations, and executing the code. Through testing across 40 varied sequencing-based analysis scenarios, AutoBA with GPT-4 attained a 65% success rate in end-to-end automation[95]. Error message feedback for code correction significantly enhanced this success rate. In addition, AutoBA utilizes retrieval-augmented generation (RAG) to increase robustness of code generation[95].

Mergen[96] is an R package that automates data analysis through LLM utilization. It crafts, executes, and refines code based on user-provided textual descriptions. The inclusion of file headers in prompts and error message feedback notably improves coding efficacy. The evaluation tasks for Mergen, while relevant to bioinformatics, cater to a general-purpose scope, covering machine learning, statistics, visualization, and data wrangling. Interestingly, the adoption of role-playing does not yield significant enhancements, possibly due to the general nature of the tasks and the mismatch between the assumed bioinformatician role and the task requirements.

LLMs exhibit inherent limitations in coding with tools beyond their training datasets. Bioinformaticians typically consult user manuals and source code to master new tools, a process LLMs could emulate. The BioMANIA framework[97] exemplifies this approach by creating conversational chatbots for open-source, well-documented Python tools. By understanding APIs from source code and user manuals, it employs GPT-4 to generate instructions for API usage. These instructions inform a BERT-based model to suggest top appropriate APIs based on a user’s query, with GPT-4 predicting parameters and executing API calls. Evaluation of the method identifies areas for improvement, such as tutorial documentation and API design, guiding the future development of chatbot-compatible tools[97].

### 7.2. BIOMEDICAL DATABASE ACCESS

Structured Query Language (SQL) serves as a pivotal tool for navigating bioinformatics databases. Mastering SQL requires users to have both programming skills and a deep understanding of the database’s data schema—prerequisites that many biomedical scientists find challenging. Recent advancements have seen LLM-chatbots like ChatGPT stepping in to translate natural language questions into SQL queries[98], significantly easing database access for non-programmers.

The work bySima and de Farias [99] explored ChatGPT-4’s ability to explain and generate SPARQL queries for public biological and bioinformatics databases. Faced with explaining a complex SPARQL query that identifies human genes linked to cancer and their orthologs in rat brains—requiring to combine data from Uniprot, OMA, and Bgee databases—ChatGPT adeptly breaked down the query’s elements. However, its attempt to craft a SPARQL query from a natural language description for the same database search revealed inaccuracies that require specific human feedback for correction. Notably, prompts augmented with sematic clues such as variable names and inline comments indicate a substantial improvement in the performance on translating questions into corresponding SPARQL queries, when evaluated on a fine-tuned OpenLlama LLM[100].

Another work by Chen and Stadler [101] applied GPT-3.5 and GPT-4 to convert user inputs into SQL queries for accessing a database of SARS-CoV-2 genomes and their annotations. Through systematic prompting and learning from numerous examples, the chatbot shows proficiency in understanding the database structure and generates accurate queries for 90.6% and 75.2% of the requests with GPT-4 and GPT-3.5, respectively. In addition, the chatbot initiates a new session to explain each query for the users to cross-ref with their own inputs to minimize risks of misunderstandings.

### 7.3. ONLINE TOOLS FOR CODING WITH CHATGPT

The Code Interpreter, officially integrated into ChatGPT-4 during the summer of 2023, represents a significant advancement in streamlining computational tasks. This feature facilitates a wide array of operations, including data upload, specification of analysis requirements, generation and execution of Python code, visualization of results, and data download, all through natural language instructions. It stands out for its ability to dynamically adapt code in response to runtime errors and self-assess the outcomes of code execution. Despite its broad applicability for general-purpose tasks such as data manipulation and visualization, its utility in bioinformatics data analysis encounters limitations such as the absence of bioinformatics-specific packages and the inability to access external databases[102].

Shortly after the release of ChatGPT in November 2022, RTutor.AI emerged as a pioneering web-server powered by the GPT technology dedicated to data analysis. This R-based platform simplifies the process for users to upload a single tabular dataset and articulate their data analysis requirements in natural language. RTutor.AI proficiently manages data importing and type conversion, subsequently leveraging OpenAI’s API for R code generation. It executes the generated code and produces downloadable HTML reports including figure plots. A subsequent application, Chatlize.AI, developed by the same team, adopts a tree-of-thought methodology[46] to enhance data analysis exploration. This approach, extending to Python, enables the generation of multiple code versions for a given analysis task, their execution, and comprehensive documentation of the results. Users benefit from the flexibility to select a specific code for further analysis. This feature is particularly valuable for exploratory data analysis, making Chatlize.AI a flexible solution for practicing prompt bioinformatics.

### 7.4. BENCHMARKS FOR BIOINFORMATICS CODING

A thorough assessment of bioinformatics necessitates the establishment of comprehensive benchmarks to cover a broad range of topics in the field. Writing individual functions is a fundamental skill in the development of advanced bioinformatics algorithms. BIOCODER[103] is a benchmark to evaluate language models’ proficiency in function writing. This benchmark encompasses over 2,200 Python and Java functions derived from authentic bioinformatics codebases, in addition to 253 functions sourced from the Rosalind project. Comparative analyses have shown that GPT-3.5 and GPT-4 significantly outperform smaller, coding-specific language models. Interestingly, integrating topic-specific context, such as imported objects, into the baseline task descriptions markedly enhances accuracy. However, even the most adept models, namely the GPT series, reach an accuracy ceiling at 60%. A significant proportion of the failures are attributed to syntax or runtime errors[103], suggesting that ChatGPT’s effectiveness in bioinformatics coding can be further enhanced through human feedback on error messages.

Execution success is crucial, yet it represents only one facet of evaluating bioinformatics code quality. Sarwal, Munteanu [104] proposed a comprehensive evaluation framework that encompassed seven metrics, assessing both subjective and objective dimensions of code writing. These dimensions include readability, correctness, efficiency, simplicity, error handling, code examples, and clarity of input/output specifications. Each metric is scaled from 1 to 10 and normalized independently post-evaluation across models. When applied to a variety of common bioinformatics tasks, this framework highlighted GPT-4’s superior performance over alternatives such as BARD and LLaMA. However, the current evaluation remains narrowly focused on a limited set of tasks[104]. Expanding these evaluations to encompass a broader range of bioinformatics domains asks for community-led efforts for a comprehensive appraisal of these language models.

## 8. CHATBOTS IN BIOINFORMATICS EDUCATION

The potential of integrating LLMs into bioinformatics education has attracted significant discussions. ChatGPT-3.5 achieves impressive performance in addressing Python programming exercises in an entry-level bioinformatics course[12]. Beyond mere code generation, the utility of chatbots extends to proposing analysis plans, enhancing code readability, elucidating error messages, and facilitating language translation in coding tasks[105]. The effectiveness of a chatbot’s response depends on the precision of human instructions, or prompts. In this context, Shue et al.[10] introduced the OPTIMAL model, which refines prompts through iterative interactions with a chatbot, mirroring the learning curve of bioinformatics novices assisted by such technologies. To navigate this evolving educational landscape, it becomes imperative to establish guidelines that enable students to critically assess outcomes and articulate constructive feedback to the chatbot for code improvement. Error messages, as one form of such feedback, turn out to be an effective way to boost the coding efficiency of ChatGPT across various studies[10, 11, 96].

The convenience of using chatbots for coding exercises poses a risk of fostering AI overreliance, leading to a superficial understanding of the underlying concepts[12, 13, 106]. This AI reliance could undermine students’ performance in summative assessments[12]. Innovative evaluation strategies, such as generating multiple-choice questions from student-submitted code to gauge their understanding[107], are needed to counteract this challenge. Such methodologies should aim to deepen students’ grasp of the material, ensuring their in-depth understanding of coding concepts.

The art of crafting effective prompts emerges as a critical skill that complements traditional programming competencies. Feedback from a pilot study involving graduate students interacting with ChatGPT for coding highlights the challenges in generating impactful prompts[108]. This prompt-related psychological strain may discourage students from using the chatbot[13]. Instructors need to equip students with prompting skills known to enhance coding efficacy, such as one/few-shot by providing examples and stimulus prompting by listing function/package names[13]. In addition, the development of a repository featuring carefully crafted prompts, accompanied by quality metrics, reference code, and outcomes, could serve as a valuable resource for students to learn coding through prompting[10, 13].

In conclusion, while chatbots demonstrate potential as educational tools, their efficacy and effectiveness have not yet been systematically evaluated in classroom settings with controlled experiments. The use of chatbots should be viewed as supplementary to traditional education methodologies[10, 12, 13]. Meanwhile, new assessment methodologies are needed to measure the pedagogical value of chatbots in enhancing bioinformatics learning without diminishing the depth of understanding of concepts and analytical skills.

## 9. DISCUSSION AND FUTURE PERSPECTIVES

The year 2023 marked significant progress in leveraging ChatGPT for bioinformatics and biomedical informatics. Early studies affirming its capability in drafting workable code for basic bioinformatics data analysis[10, 12]. The chatbot has also demonstrated competitiveness with SOTA models in other bioinformatics areas, including identifying cell type from single-cell RNA-Seq data[16], performing question-answering tasks in biomedical text mining[26], and generating molecular captions in drug discovery[52]. These achievements underscore ChatGPT’s proficiency in text-generative tasks. Meanwhile, other LLMs are catching up. For example, Google developed Gemini and open-source LLM Gemma, which delivered impressive performance in various tasks. Although their applications in bioinformatics and medical informatics have not been reported, their potentials provide users a viable alternative to ChatGPT.

Current chatbots exhibit limitations in performing biomedical tasks that require reasoning and quantitative analysis, such as regression and classification, as evidenced by references[30, 32, 67, 68, 104]. Though not yet widely adapted in bioinformatics[18], OpenAI’s fine-tuning APIs such as for GPT-3.5 and GPT-4 hold great potential for performance improvements when the training dataset is large. Nevertheless, the accuracy of ChatGPT’s responses can be significantly improved through a strategic design of its input instructions with prompt engineering. Incorporating examples into prompts and employing CoT reasoning has proven an effective strategy, as evidenced in various bioinformatics applications[30, 35, 62, 67, 68, 101]. While examples in prompts are sometimes hardcoded, they can also be dynamically and strategically sourced from external knowledge bases or knowledge graphs[62, 63, 65, 109]. This approach, known as retrieval-augmented generation (RAG), improves ChatGPT’s reliability by sourcing facts from domain-specific knowledge and represents a promising avenue for future bioinformatics with chatbots.

In this rapidly evolving domain, ChatGPT has experienced several significant upgrades within its first year alone. We acknowledge that not every upgrade enhances performance across the board[110]. Consequently, prompts that are highly effective with the current version for specific tasks may not maintain the same level of efficacy following future updates. The technique of prompt engineering, which includes strategies like role prompting and in-context learning, offers a way to partially counteract this variability^[45]^. An innovative approach, rather than manually adjusting the prompts, involves instructing ChatGPT to autonomously optimize prompts to align with its latest model iteration. This strategy has shown promise in tasks such as mining gene relationships[45] but remains largely unexplored in other bioinformatics topics and therefore warrants further exploration to fully leverage ChatGPT’s capabilities in the field.

Numerous studies repeatedly show that using ChatGPT with human augmentations significantly improve the performance. Iterative human-AI communication plays a pivotal role in this process, where feedback from human operator grounds the chatbot’s responses for improved accuracy. This human-in-the-loop methodology is particularly evident in prompt optimization[10] and molecular optimization[60, 63]. For code generation tasks, runtime error message represents commonly used feedback that has been automated into several GPT-based tools[95, 96, 102]. Conversely, the chatbot can also be instructed to provide feedback to human operators. As demonstrated byChen and Stadler [101], ChatGPT can produce textual descriptions for the generated code through an inverse generation process. Comparing these descriptions with the original instructions from the human operator ensures that the chatbot’s output aligns closely with the intended task requirements. This iterative exchange of feedback between AI and human operators enhances the overall quality of the bioinformatics tasks being addressed.

The assessment of ChatGPT’s capabilities across various bioinformatics tasks has illuminated both its strengths and weaknesses. However, the reliability of these evaluations largely hinges on the quality of the benchmarks used and the methodologies applied in these assessments. Currently, many benchmarks are available for biomedical text mining and chemistry-related tasks. The development of benchmarks designed specifically for assessing ChatGPT’s capability in other bioinformatics tasks, including multimodality, is still in its infancy. It’s important to recognize that in generative tasks like coding, producing expected results is not the sole criterion for gauging effectiveness and efficiency. Factors such as the readability of the code and the inclusion of code examples also play crucial roles[104]. Nonetheless, conducting such comprehensive evaluations can be resource-intensive, underscoring the need for community efforts to enhance the scope. While alternatives exist for automation, such as transforming tasks into multiple-choice questions or verifying responses against reference texts, for example through lexical overlap or semantic similarity, each method comes with its own set of limitations[7]. Consequently, there is a pressing need to develop new, scalable, and accurate evaluation metrics and benchmark datasets that can accommodate a wide range of bioinformatics tasks, ensuring that assessments are both meaningful and reflective of real-world and cutting-edge applicability.

While aiming for comprehensiveness, our review does not encompass areas that, although outside the direct scope of bioinformatics and biomedical informatics, are closely related and significant. These areas include the management of electronic health records[111, 112], emotion analysis through social media[113], and medical consultation[114, 115]. To mitigate transparency and security concerns, fine-tuning open-source language models deployed locally with task-specific instructions presents a practical approach. Our review has spotlighted such advancements for drug discovery. However, we refer our readers to additional reviews for an expansive understanding of similar developments in other bioinformatics topics, as well as the ethical and legal issues involved[7–9, 116, 117]. Looking ahead, we envision a future where both online proprietary models such as ChatGPT and open-source, locally deployable finetuned language models coexist for bioinformatics and biomedical informatics, ensuring users with the most suitable tools to address their specific needs.

## Figures and Tables

**Table 1.** Terminologies cited in this review.

Term	Definition
Prompt engineering	The practice of designing and refining input prompts (natural language instruction) to elicit desired responses from a language model chatbot.
Zero-shot	A way of prompting where instruction to the chatbot contains no example of a specified task.
One-shot	A way of prompting where instruction to the chatbot contains one example of a specified task.
Few-shot	A way of prompting where instruction to the chatbot contains more than one examples of that task.
Chain of Thought (CoT)	A way of prompting asking the chatbot to think step by step. This approach helps in enhancing the model’s ability to solve complex problems by breaking them down into simpler, sequential steps. For one/few-shot, if an example includes details of step-by-step reasoning, the example is called CoT example.
Tree of Thought (ToT)	An extension of the Chain of Thought approach, where the model generates a tree-like structure of reasoning steps instead of a linear chain.
In-Context Learning (ICL)	A learning paradigm where a model leverages the context provided within the input to adapt and respond to new tasks or information without explicit retraining.
Retrieval-Augmented Generation (RAG)	A technique that combines a retriever model, which fetches relevant documents or data, with a generator model, which uses the retrieved information to generate responses or complete tasks. This approach is useful for tasks that require external knowledge or context.
Fine-tuning	The process of further training a pre-trained model on a specific dataset or task to improve its performance in that area.
Instruction tuning	The process of fine-tuning a pre-trained model to better understand and follow natural language instructions, improving its applicability across different tasks.
Task tuning	The process of fine-tuning a pre-trained model on a specific task to enhance its performance on that task.
AI hallucination	The phenomenon where a generative AI model produces false or misleading information not supported by the input data or its training.
