# Supplementary material for: Bioinformatics and biomedical informatics with ChatGPT: Year one review
Source: ArXiv. 2024 Jun 12:arXiv:2403.15274v2. Originally published 2024 Mar 22. Preprint. [Version 2] (PMC10984005)
Supplement: Supplement 1 [file NIHPP2403.15274v2-supplement-1.pdf]

# Supplementary Table S1: List of studies discussed in this review.

| Areas | Manuscript Title | Preprint | Journal/Conference | Date of online release | Github | Category |
|---|---|---|---|---|---|---|
| Bioinformatics programming | On the Potential of Artificial Intelligence Chatbots for Data Exploration of Federated Bioinformatics Knowledge Graphs | Arxiv | SeWebMeDa'23: 6th Workshop on Semantic Web solutions for large-scale biomedical data analytics | April 20, 2023 | NA | Evaluation |
| Bioinformatics programming | AI chatbots can boost scientific coding | NA | Nature Ecology & Evolution | April 26, 2023 | NA | Evaluation |
| Bioinformatics programming | Code interpreter for bioinformatics: are we there yet? | NA | Annals of Biomedical Engineering | July 23, 2023 | NA | Evaluation |
| Bioinformatics programming | Biocoder: A benchmark for bioinformatics code generation with contextual pragmatic knowledge | Arxiv | Bioinformatics (in press) | August 31, 2023 | https://github.com/gersteinlab/biocoder | Evaluation |
| Bioinformatics programming | SPARQL Generation: an analysis on fine-tuning OpenLLaMA for Question Answering over a Life Science Knowledge Graph | Arxiv | SWAT4HCLS 2024: The 15th International Conference on Semantic Web Applications and Tools for Health Care and | February 7, 2024 | https://github.com/RIKEN-DKO/Generation_SPARQL | Evaluation |
| Biomedical image understanding | Scientific figures interpreted by chatgpt: Strengths in plot recognition and limits in color perception | BioRxiv | npj Precision Oncology | October 17, 2023 | NA | Evaluation |
| Biomedical image understanding | A pilot study on the efficacy of GPT-4 in providing orthopedic treatment recommendations from MRI reports | NA | Scientific Reports | November 17, 2023 | NA | Evaluation |
| Biomedical text mining | Evaluation of ChatGPT Family of Models for Biomedical Reasoning and Classification | Arxiv | JAMIA | April 5, 2023 | https://github.com/shan23chen/HealthLLM_Eval | Evaluation |
| Biomedical text mining | An extensive benchmark study on biomedical text generation and mining with chatgpt. | BioRxiv | Bioinformatics | April 20, 2023 | NA | Evaluation |
| Biomedical text mining | Opportunities and Challenges for ChatGPT and Large Language Models in Biomedicine and Health | Arxiv | Briefings in Bioinformatics | June 15, 2023 | NA | Evaluation |
| Biomedical text mining | Is ChatGPT a Biomedical Expert?--Exploring the Zero-Shot Performance of Current GPT Models in Biomedical Tasks | Arxiv | 11th BioASQ Workshop at CLEF 2023 | June 28, 2023 | https://github.com/SamyAteia/bioasq | Evaluation |
| Biomedical text mining | A comprehensive evaluation of large language models on benchmark biomedical text processing tasks | Arxiv | Computers in Biology and Medicine journal | October 6, 2023 | https://github.com/tahmedge/llm-eval-biomed | Evaluation |
| Biomedical text mining | Iterative Prompt Refinement for Mining Gene Relationships from ChatGPT | BioRxiv | International Journal of Artificial Intelligence and Robotics Research (in press) | December 23, 2023 | NA | Evaluation |
| Biomedical text mining | EVLncRNAs 3.0: an updated comprehensive database for manually curated functional long non-coding RNAs validated by low-throughput experiments | NA | Nucleic Acids Research | January 1, 2024 | https://www.sdklab-biophysics-dzu.net/EVLncRNAs3/#/ | Application |
| Biomedical text mining | Computational screening of biomarkers and potential drugs for arthrofibrosis based on combination of sequencing and large nature language model | NA | Journal of Orthopaedic Translation | January 20, 2024 | https://chenxi2023.shinyapps.io/afdbv1/ | Application |
| Biomedical text mining | A Comprehensive Evaluation of Large Language Models in Mining Gene Interactions and Pathway Knowledge | BioRxiv | Quantitative Biology (in press) | January 24, 2024 | https://github.com/Muh-aza/LLM | Evaluation |
| Chatbots in bioinformatics education | Evaluating a large language model's ability to solve programming exercises from an introductory bioinformatics course | aRxiv | PLoS Computational Biology | March 7, 2023 | NA | Evaluation |
| Chatbots in bioinformatics education | Empowering Beginners in Bioinformatics with ChatGPT | BioRxiv | Quantitative Biology | March 8, 2023 | NA | Evaluation |
| Drug discovery | The capability of ChatGPT in predicting and explaining common drug-drug interactions | NA | Cureus | March 17, 2023 | NA | Evaluation |
| Drug discovery | ChatGPT-powered Conversational Drug Editing Using Retrieval and Domain Feedback | Arxiv | ICLR 2024 | May 18, 2023 | https://github.com/chao1224/ChatDrug | Application |
| Drug discovery | What can large language models do in chemistry? a comprehensive benchmark on eight tasks | Arxiv | Advances in Neural Information Processing Systems | May 27, 2023 | https://github.com/ChemFoundationModels/ChemLLMBench | Evaluation |
| Drug discovery | Empowering molecule discovery for molecule-caption translation with large language models: A chatgpt perspective | Arxiv | IEEE TRANSACTIONS ON KNOWLEDGEANDDATAENGINEERING (in press) | June 11, 2023 | https://github.com/phenixace/MolReGPT | Evaluation |
| Drug discovery | Mol-instructions: A large-scale biomolecular instruction dataset for large language models | Arxiv | ICLR 2024 | June 13, 2023 | https://github.com/zjunlp/Mol-Instructions | Evaluation |
| Drug discovery | Performance of ChatGPT on the pharmacist licensing examination in Taiwan | NA | Journal of the Chinese Medical Association | July 5, 2023 | NA | Evaluation |
| Drug discovery | Performance of ChatGPT on Chinese national medical licensing examinations: a five-year examination evaluation study for physicians, pharmacists and nurses | medRxiv | BMC Medical Education | August 02, 2023 | NA | Evaluation |
| Drug discovery | Evaluating the performance of ChatGPT in clinical pharmacy: a comparative study of ChatGPT and clinical pharmacists | NA | British journal of clinical pharmacology | August 25, 2023 | NA | Evaluation |
| Drug discovery | Evaluating the sensitivity, specificity, and Accuracy of ChatGPT-3.5, ChatGPT-4, Bing AI, and bard against conventional drug-drug interactions clinical tools | NA | Drug, Healthcare and Patient Safety | September 20, 2023 | NA | Evaluation |
| Drug discovery | Leveraging large language models for predictive chemistry | ChemRxiv | Nature Machine Intelligence | October 17, 2023 | https://github.com/kjappelbaum/gptchem | Evaluation |
| Drug discovery | Examining the Potential of ChatGPT on Biomedical Information Retrieval: Fact-Checking Drug-Disease Associations | NA | Annals of Biomedical Engineering | October 19, 2023 | NA | Evaluation |
| Drug discovery | A generative drug–drug interaction triplets extraction framework based on large language models | NA | Proceedings of the Association for Information Science and Technology | October 22, 2023 | | Evaluation |
| Drug discovery | The potential of GPT-4 as a support tool for pharmacists: analytical study using the Japanese national examination for pharmacists | NA | JMIR Medical Education | October 30, 2023 | NA | Evaluation |
| Drug discovery | Fine-tuning large language models for chemical text mining | ChemRxiv | Chemical Science (in press) | November 16, 2023 | https://github.com/zw-SIMM/SFTChatGPT_for_chemtext_mining | Evaluation |
| Drug discovery | ChatGPT in Drug Discovery: A Case Study on Anticocaine Addiction Drug Development with Chatbots | NA | Journal of Chemical Information and Modeling | November 13, 2023 | https://github.com/wangru25/SGNC | Application |
| Drug discovery | Comprehensive evaluation of molecule property prediction with ChatGPT | NA | Methods | January 17, 2024 | NA | Evaluation |

| | | | | | | |
|---|---|---|---|---|---|---|
| Genetics | Analysis of large-language model versus human performance for genetics questions | medRxiv | European Journal of Human Genetics | January 28, 2023 | NA | Evaluation |
| Genetics | Can chatgpt understand genetics? | NA | European Journal of Human Genetics | July 5, 2023 | NA | Evaluation |
| Genetics | Is artificial intelligence getting too much credit in medical genetics? | NA | American Journal of Medical Genetics | August 22, 2023 | NA | Evaluation |
| Genetics | Genetic counselors' utilization of ChatGPT in professional practice: A cross-sectional study | NA | American Journal of Medical Genetics | December 8, 2023 | NA | Evaluation |
| Omics | Assessing GPT-4 for cell type annotation in single-cell RNA-seq analysis | BioRxiv | Nature Methods | April 21, 2023 | https://github.com/Winnie09/GPTCelltype_Paper | Evaluation |
| Omics | Genegpt: Augmenting large language models | Arxiv | Bioinformatics | May 16, 2023 | https://github.com/ncbi/GeneGPT. | Application |
| Bioinformatics programming | GenSpectrum Chat: Data Exploration in Public Health Using Large Language Models | Arxiv | NA | May 23, 2023 | https://cov-spectrum.org/chat | Application |
| Bioinformatics programming | BioMANIA: Simplifying bioinformatics data analysis through conversation | BioRxiv | NA | November 1, 2023 | https://github.com/batmen-lab/BioMANIA | Application |
| Bioinformatics programming | Leveraging large language models for data analysis automation | BioRxiv | NA | December 21, 2023 | https://github.com/BIMSBbioinfo/mergen-manuscript | Application |
| Bioinformatics programming | An AI Agent for Fully Automated Multi-omic Analyses | BioRxiv | NA | January 5, 2024 | https://github.com/JoshuaChou2018/AutoBA | Application |
| Bioinformatics programming | BioLLMBench: A Comprehensive Benchmarking of Large Language Models in Bioinformatics | BioRxiv | NA | January 16, 2024 | NA | Evaluation |
| Biomedical image understanding | Accuracy of a vision-language model on challenging medical cases | Arxiv | NA | November 9, 2023 | NA | Evaluation |
| Biomedical image understanding | Performance of multimodal gpt-4v on usmle with image: Potential for imaging diagnostic support with explanations | medRxiv | NA | November 15, 2023 | NA | Evaluation |
| Biomedical image understanding | GPT-4V exhibits human-like performance in biomedical image classification | BioRxiv | NA | January 1, 2024 | https://github.com/Winnie09/gptimage | Evaluation |
| Biomedical image understanding | Hidden Flaws Behind Expert-Level Accuracy of GPT-4 Vision in Medicine | Arxiv | NA | January 16, 2024 | NA | Evaluation |
| Biomedical text mining | Evaluation of GPT and BERT-based models on identifying protein-protein interactions in biomedical text | Arxiv | NA | March 30, 2023 | NA | Evaluation |
| Biomedical text mining | Large language models in biomedical natural language processing: benchmarks, baselines, and recommendations | Arxiv | NA | May 10, 2023 | https://github.com/qingyu-qc/gpt_bionlp_benchmark | Evaluation |
| Biomedical text mining | PlantConnectome: knowledge networks encompassing> 100,000 plant article abstracts | BioRxiv | NA | July 15, 2023 | https://connectome.plant.tools/ | Application |
| Biomedical text mining | From answers to insights: Unveiling the strengths and limitations of chatgpt and biomedical knowledge graphs | Res Sq | NA | August 1, 2023 | NA | Evaluation |
| Biomedical text mining | ChatGPT usage in the Reactome curation process | BioRxiv | NA | November 8, 2023 | NA | Application |
| Biomedical text mining | reguloGPT: Harnessing GPT for Knowledge Graph Construction of Molecular Regulatory Pathways | BioRxiv | NA | January 30, 2024 | https://github.com/Huang-AI4Medicine-Lab/reguloGPT | Application |
| Drug discovery | Bayesian optimization of catalysts with in-context learning | Arxiv | NA | April 11, 2023 | https://github.com/ur-whitelab/BO-LIFT | Evaluation |
| Drug discovery | DrugChat: towards enabling ChatGPT-like capabilities on drug molecule graphs | Arxiv | NA | May 18, 2023 | https://github.com/UCSD-AI4H/drugchat | Application |
| Drug discovery | Interactive molecular discovery with natural language | Arxiv | NA | June 21, 2023 | https://github.com/Ellenzzn/ChatMol/tree/main | Application |
| Drug discovery | Instructmol: Multi-modal integration for building a versatile and reliable molecular assistant in drug discovery | Arxiv | NA | November 27, 2023 | https://idea-xl.github.io/InstructMol/ | Application |
| Drug discovery | Drugassist: A large language model for molecule optimization | Arxiv | NA | December 28, 2023 | https://github.com/blazerye/DrugAssist | Application |
| Drug discovery | ChemDFM: Dialogue Foundation Model for Chemistry | Arxiv | NA | January 26, 2024 | NA | Application |
| Omics | GeneTuring tests GPT models in genomics | BioRxiv | NA | March 13, 2023 | NA | Evaluation |

For preprints not yet formally published, those cited to support shared findings across independent works are underlined.

# Supplementary Table S2: Performance comparison of ChatGPT to baseline models on biomedical text mining tasks.

| | | | | ChatGPT | | | | | | | | BERT | | | | | | Alternative LLMs to ChatGPT | | | | | | | | | | | | | | | | | | | Others |
|---|---|---|---|---|---|---|---|---|---|---|---|---|---|---|---|---|---|---|---|---|---|---|---|---|---|---|---|---|---|---|---|---|---|---|---|---|---|
| Reference Title | Tasks | Benchmark | Evaluation Metrics | GPT-3.5 | 3.5 (one-shot) | 3.5 (few-shot) | 3.5 (few-shot | GPT-4 | GPT-4 (one-shot) | GPT-4 (few-shot) | (few-shot CoT) | PubMedBERT | BioLinkBERT-Base | BioLinkBERT-Large | BioBERT | PubmedBert | SciBERT | Claude-2 | Claude-Instant | Cohere | Claude-Pro | PaLM-2 | Bard | Codellama-34 | Wizardlm-70b | Wizardlm-13b | Falcon-180b | Mistral-7b | Chatglm2-6b | Vicuna-7b | Vicuna-33b | Vicuna-13b | llama-2-70b | LLaMa-2-13b | llama-2-7b | Qwen-14b | BoW |
| An extensive benchmark study on biomedical text generation and mining with chatgpt. | Named entity recognition | BC5-chem | F1 entity-level | 60.3 | - | - | - | - | - | - | - | 93.33 | 93.75 | 94.04 | - | - | - | - | - | - | - | - | - | - | - | - | - | - | - | - | - | - | - | - | - | - | |
| | Named entity recognition | BC5-disease | F1 entity-level | 51.77 | - | - | - | - | - | - | - | 85.62 | 86.1 | 86.39 | - | - | - | - | - | - | - | - | - | - | - | - | - | - | - | - | - | - | - | - | - | - | |
| | Named entity recognition | NCBI-disease | F1 entity-level | 50.49 | - | - | - | - | - | - | - | 87.82 | 88.18 | 88.76 | - | - | - | - | - | - | - | - | - | - | - | - | - | - | - | - | - | - | - | - | - | - | |
| | Named entity recognition | BC2GM | F1 entity-level | 37.54 | - | - | - | - | - | - | - | 84.52 | 84.9 | 85.18 | - | - | - | - | - | - | - | - | - | - | - | - | - | - | - | - | - | - | - | - | - | - | |
| | Named entity recognition | JNLPBA | F1 entity-level | 41.25 | - | - | - | - | - | - | - | 80.06 | 79.03 | 80.06 | - | - | - | - | - | - | - | - | - | - | - | - | - | - | - | - | - | - | - | - | - | - | |
| | PICO extraction | EBM PICO | Macro F1 word-level | 55.59 | - | - | - | - | - | - | - | 73.38 | 73.97 | 74.19 | - | - | - | - | - | - | - | - | - | - | - | - | - | - | - | - | - | - | - | - | - | - | |
| | Relation extraction | ChemProt | Micro F1 | 34.16 | 48.64 | - | - | - | | - | - | 77.24 | 77.57 | 79.98 | - | - | - | - | - | - | - | - | - | - | - | - | - | - | - | - | - | - | - | - | - | - | |
| | Relation extraction | DDI | Micro F1 | 51.62 | - | - | - | - | - | - | - | 82.36 | 82.72 | 83.35 | - | - | - | - | - | - | - | - | - | - | - | - | - | - | - | - | - | - | - | - | - | - | |
| | Relation extraction | GAD | Micro F1 | 52.43 | - | - | - | - | - | - | - | 82.34 | 84.39 | 84.9 | - | - | - | - | - | - | - | - | - | - | - | - | - | - | - | - | - | - | - | - | - | - | |
| | Sentence similarity | BIOSSES | Pearson | 43.75 | - | - | - | - | - | - | - | 92.3 | 93.25 | 93.63 | - | - | - | - | - | - | - | - | - | - | - | - | - | - | - | - | - | - | - | - | - | - | |
| | Document classification | HoC | Average Micro F1 | 51.22 | - | - | - | - | - | - | - | 82.34 | 84.39 | 84.9 | - | - | - | - | - | - | - | - | - | - | - | - | - | - | - | - | - | - | - | - | - | - | |
| | Question answering | PubMedQA | Accuracy | 76.45 | - | - | - | - | - | - | - | 55.84 | 70.2 | 72.18 | - | - | - | - | - | - | - | - | - | - | - | - | - | - | - | - | - | - | - | - | - | - | |
| | Question answering | BioASQ | Accuracy | 88.57 | - | - | - | - | - | - | - | 87.56 | 91.43 | 94.82 | - | - | - | - | - | - | - | - | - | - | - | - | - | - | - | - | - | - | - | - | - | - | |
| Large language models in biomedical natural language processing: benchmarks, baselines, and recommendations | Named entity recognition | BC5CDR-chemical | F1 entity-level | 68.36 | 72.1 | - | - | 81.9 | 82.43 | - | - | 93.5 | - | - | - | - | - | - | - | - | - | - | - | - | - | - | - | - | - | - | - | - | - | - | - | - | |
| | Named entity recognition | NCBI-disease | F1 entity-level | 38.02 | 42.74 | - | - | 57.85 | 58.39 | - | - | 89.86 | - | - | - | - | - | - | - | - | - | - | - | - | - | - | - | - | - | - | - | - | - | - | - | - | |
| | Relation extraction | ChemProt | Micro F1 | 57.43 | 57.71 | - | - | 66.18 | 66.82 | - | - | 78.32 | - | - | - | - | - | - | - | - | - | - | - | - | - | - | - | - | - | - | - | - | - | - | - | - | |
| | Relation extraction | DDI2013 | Micro F1 | 33.49 | 34.34 | - | - | 63.25 | 61.76 | - | - | 80.23 | - | - | - | - | - | - | - | - | - | - | - | - | - | - | - | - | - | - | - | - | - | - | - | - | |
| | Multi-label classification | HoC | Label-wise macro F1 | 65.72 | 69.32 | - | - | 74.74 | 74.02 | - | - | 89.15 | - | - | - | - | - | - | - | - | - | - | - | - | - | - | - | - | - | - | - | - | - | - | - | - | |
| | Multi-label classification | LitCovid | Label-wise macro F1 | 63.9 | 65.31 | - | - | 67.46 | 68.39 | - | - | 87.24 | - | - | - | - | - | - | - | - | - | - | - | - | - | - | - | - | - | - | - | - | - | - | - | - | |
| | Semantic similarity and reasoning | PubMedQA | Pearson | 35.53 | 30.11 | - | - | 43.74 | 53.61 | - | - | 36.76 | - | - | - | - | - | - | - | - | - | - | - | - | - | - | - | - | - | - | - | - | - | - | - | - | |
| | Semantic similarity and reasoning | BIOSSES | Pearson | 87.86 | 91.94 | - | - | 88.32 | 89.22 | - | - | 93.32 | - | - | - | - | - | - | - | - | - | - | - | - | - | - | - | - | - | - | - | - | - | - | - | - | |
| | Text summarization | summarization | ROUGE-1 | 6.08 | 23.2 | - | - | 39.97 | 40.54 | - | - | 44.89 | - | - | - | - | - | - | - | - | - | - | - | - | - | - | - | - | - | - | - | - | - | - | - | - | |
| | Text summarization | MS^2 | ROUGE-1 | 17.31 | 12.11 | - | - | 18.77 | 19.19 | - | - | 20.79 | - | - | - | - | - | - | - | - | - | - | - | - | - | - | - | - | - | - | - | - | - | - | - | - | |
| | Text simplification | Cochrane PLS | Flesch-Kincaid score | 13.0505 | 13.18 | - | - | 12.0001 | 13.12 | - | - | 12.64 | - | - | - | - | - | - | - | - | - | - | - | - | - | - | - | - | - | - | - | - | - | - | - | - | |
| | Text simplification | PLOS text simplification | Flesch-Kincaid score | 14.0605 | 13.92 | - | - | 13.219 | 13.24 | - | - | 14.66 | - | - | - | - | - | - | - | - | - | - | - | - | - | - | - | - | - | - | - | - | - | - | - | - | |
| Evaluation of ChatGPT Family of Models for Biomedical Reasoning and Classification | Classification | "Advice in discussion sections" | Micro F1 | 50.6 | 51.3 | 47.5 | 67.1 | 50.9 | - | - | 64.8 | - | - | - | 80 (100% fine-tuning) | - | - | - | - | - | - | - | - | - | - | - | - | - | - | - | - | - | - | - | - | - | 59.3 (100% fine-tuning) |
| | Classification | "Advice in unstructured abstracts" | Micro F1 | 48.9 | 55.4 | 40.3 | 67 | 47.8 | - | - | 71.2 | - | - | - | 82.1 (100% fine-tuning) | - | - | - | - | - | - | - | - | - | - | - | - | - | - | - | - | - | - | - | - | - | 64 (100% fine-tuning) |
| | Classification | "Advice in structured abstracts" | Micro F1 | 54.8 | 59.3 | 49.5 | 71.8 | 51.7 | - | - | 77 | - | - | - | 90.2 (100% fine-tuning) | - | - | - | - | - | - | - | - | - | - | - | - | - | - | - | - | - | - | - | - | - | 75.3 (100% fine-tuning) |
| | Reasoning | "Causal relation detection" | Micro F1 | 28.8 | 39.6 | 54.2 | 64.9 | 30.1 | - | - | 68.2 | - | - | - | 85.1 (100% fine-tuning) | - | - | - | - | - | - | - | - | - | - | - | - | - | - | - | - | - | - | - | - | - | 67.5 (100% fine-tuning) |
| A comprehensive evaluation of large language models on benchmark biomedical text processing tasks | Relation extraction | BC5CDR | F1 | 43.29 | - | - | - | - | - | - | - | - | - | - | - | - | - | 53.37 | - | - | - | 54.3 | - | - | - | - | - | - | - | - | - | - | - | 53.28 | - | - | - |
| | Relation extraction | KD-DTI | F1 | 29.74 | - | - | - | - | - | - | - | - | - | - | - | - | - | 28.84 | - | - | - | 38.44 | - | - | - | - | - | - | - | - | - | - | - | 24.21 | - | - | - |
| | Relation extraction | DDI | F1 | 46.43 | - | - | - | - | - | - | - | - | - | - | - | - | - | 42.62 | - | - | - | 22.5 | - | - | - | - | - | - | - | - | - | - | - | 24.03 | - | - | - |
| | Text classification | HoC | F1 | 59.26 | - | - | - | - | - | - | - | - | - | - | - | - | - | 34.93 | - | - | - | 61.03 | - | - | - | - | - | - | - | - | - | - | - | 41.82 | - | - | - |
| | Text classification | LitCovid | F1 | 29.63 | - | - | - | - | - | - | - | - | - | - | - | - | - | 7.6 | - | - | - | 37.5 | - | - | - | - | - | - | - | - | - | - | - | 11.34 | - | - | - |
| | Question answering | PubMedQA | Accuracy | 54.4 | - | - | - | - | - | - | - | - | - | - | - | - | - | 57.2 | - | - | - | 59.6 | - | - | - | - | - | - | - | - | - | - | - | 61.4 | - | - | - |
| | Question answering | MediQA-2019 | Accuracy | 73.26 | - | - | - | - | - | - | - | - | - | - | - | - | - | 65.13 | - | - | - | 52.12 | - | - | - | - | - | - | - | - | - | - | - | 56.01 | - | - | - |
| | Entity linking dataset | BC5CDR | Recall@1 | 54.9 | - | - | - | - | - | - | - | - | - | - | - | - | - | 78.01 | - | - | - | 52.14 | - | - | - | - | - | - | - | - | - | - | - | 66.52 | - | - | - |
| | Entity linking dataset | Cometa | Recall@1 | 43.45 | - | - | - | - | - | - | - | - | - | - | - | - | - | 53.29 | - | - | - | 48.76 | - | - | - | - | - | - | - | - | - | - | - | 40.67 | - | - | - |
| | Entity linking dataset | NCBI | Recall@1 | 52.19 | - | - | - | - | - | - | - | - | - | - | - | - | - | 70.21 | - | - | - | 38.44 | - | - | - | - | - | - | - | - | - | - | - | 59.17 | - | - | - |
| | Named entity recognition | BC2GM | F1 | 31.99 | - | - | - | - | - | - | - | - | - | - | - | - | - | 40.45 | - | - | - | 32.75 | - | - | - | - | - | - | - | - | - | - | - | 5.95 | - | - | - |
| | Named entity recognition | BC4CHEMD | F1 | 26.01 | - | - | - | - | - | - | - | - | - | - | - | - | - | 35.18 | - | - | - | 25.83 | - | - | - | - | - | - | - | - | - | - | - | 6.64 | - | - | - |
| | Named entity recognition | BC5CDR-chem | F1 | 41.25 | - | - | - | - | - | - | - | - | - | - | - | - | - | 58.05 | - | - | - | 48.08 | - | - | - | - | - | - | - | - | - | - | - | 12.21 | - | - | - |
| | Named entity recognition | BC5CDR-disease | F1 | 32.26 | - | - | - | - | - | - | - | - | - | - | - | - | - | 50.13 | - | - | - | 33.72 | - | - | - | - | - | - | - | - | - | - | - | 5.68 | - | - | - |
| | Named entity recognition | JNLPBA | F1 | 31.89 | - | - | - | - | - | - | - | - | - | - | - | - | - | 34.62 | - | - | - | 21.18 | - | - | - | - | - | - | - | - | - | - | - | 4.3 | - | - | - |
| | Named entity recognition | NCBI-disease | F1 | 33.39 | - | - | - | - | - | - | - | - | - | - | - | - | - | 45.75 | - | - | - | 31.15 | - | - | - | - | - | - | - | - | - | - | - | 4.58 | - | - | - |
| | Named entity recognition | linnaeus | F1 | 5.14 | - | - | - | - | - | - | - | - | - | - | - | - | - | 13.91 | - | - | - | 6.44 | - | - | - | - | - | - | - | - | - | - | - | 1.42 | - | - | - |
| | Named entity recognition | s800 | F1 | 15.57 | - | - | - | - | - | - | - | - | - | - | - | - | - | 24.07 | - | - | - | 16.96 | - | - | - | - | - | - | - | - | - | - | - | 1.87 | - | - | - |
| | Text summarization | iCliniq | ROUGE-1 | 30.5 | - | - | - | - | - | - | - | - | - | - | - | - | - | 28.8 | - | - | - | 21.9 | - | - | - | - | - | - | - | - | - | - | - | 20 | - | - | - |
| | Text summarization | HealthCareMagic | ROUGE-1 | 28.1 | - | - | - | - | - | - | - | - | - | - | - | - | - | 24.4 | - | - | - | 25.9 | - | - | - | - | - | - | - | - | - | - | - | 16.7 | - | - | - |
| | Text summarization | MeQSum | ROUGE-1 | 30 | - | - | - | - | - | - | - | - | - | - | - | - | - | 31.7 | - | - | - | 31.5 | - | - | - | - | - | - | - | - | - | - | - | 21.2 | - | - | - |
| | Text summarization | MEDIQA-QS | ROUGE-1 | 30.6 | - | - | - | - | - | - | - | - | - | - | - | - | - | 32 | - | - | - | 29.7 | - | - | - | - | - | - | - | - | - | - | - | 23.3 | - | - | - |
| | Text summarization | MEDIQA-MAS | ROUGE-1 | 38.9 | - | - | - | - | - | - | - | - | - | - | - | - | - | 13.4 | - | - | - | 15.3 | - | - | - | - | - | - | - | - | - | - | - | 13.7 | - | - | - |
| | Text summarization | MEDIQA-ANS | ROUGE-1 | 28.7 | - | - | - | - | - | - | - | - | - | - | - | - | - | 28.6 | - | - | - | 25.4 | - | - | - | - | - | - | - | - | - | - | - | 28 | - | - | - |
| | Text summarization | PLOS (Abstract) | ROUGE-1 | 39.65 | - | - | - | - | - | - | - | - | - | - | - | - | - | 42.25 | - | - | - | 25.09 | - | - | - | - | - | - | - | - | - | - | - | 41.78 | - | - | - |
| | Text summarization | Summarization) | ROUGE-1 | 39.13 | - | - | - | - | - | - | - | - | - | - | - | - | - | 36.16 | - | - | - | 30.7 | - | - | - | - | - | - | - | - | - | - | - | 36.33 | - | - | - |
| Evaluation of GPT and BERT-based models on identifying protein-protein interactions in biomedical text | Relation extraction | LLL | F1-Score | 76.72 (with protein dictionary) | - | - | - | 86.49 (with protein dictionary) | - | - | - | - | - | - | 86.84 | 85.42 | 84.66 | - | - | - | - | - | - | - | - | - | - | - | - | - | - | - | - | - | - | - | - |
| | Relation extraction | IEPA | F1-Score | 31.25 (with protein dictionary) | - | - | - | 48.37 (with protein dictionary) | - | - | - | - | - | - | 78.81 | 78.49 | 75.53 | - | - | - | - | - | - | - | - | - | - | - | - | - | - | - | - | - | - | - | - |
| | Relation extraction | HPRD50 | F1-Score | 52.9 (with protein dictionary) | - | - | - | 57.24 (with protein dictionary) | - | - | - | - | - | - | 74.95 | 79.65 | 74.67 | - | - | - | - | - | - | - | - | - | - | - | - | - | - | - | - | - | - | - | - |
| A Comprehensive Evaluation of Large Language Models in Mining Gene Interactions and Pathway Knowledge | Relation extraction | Gene regulatory relations | F1 | 0.1485 | - | - | - | 0.4448 | - | - | - | - | - | - | - | - | - | 0.422 | 0.326 | 0.374 | 0.439 | 0.261 | 0.279 | 0.144 | 0.045 | 0.096 | 0.279 | 0.123 | 0.182 | 0.123 | 0.141 | 0.181 | 0.158 | 0.091 | 0.192 | 0.101 | - |
| | Relation extraction | KEGG pathway recognition | Jaccard similarity | 0.1358 | - | - | - | 0.2778 | - | - | - | - | - | - | - | - | - | 0.189 | 0.152 | 0.209 | 0.266 | 0.105 | 0.192 | 0.162 | 0.135 | 0.146 | 0.224 | 0.143 | 0.088 | 0.174 | 0.166 | 0.154 | 0.154 | 0.171 | 0.221 | 0.084 | - |

Values are extracted from corresponding reference listed in the first column. In red are numbers that are no better than at least one kind of GPT.

## Supplementary Table S3: Performance comparison of ChatGPT to baseline models on drug discovery tasks.

| | | | | ChatGPT | | | Baseline models | | | | | | | | | | | | | | | | | | |
|---|---|---|---|---|---|---|---|---|---|---|---|---|---|---|---|---|---|---|---|---|---|---|---|---|---|
| Reference Title | Tasks | Benchmark | Evaluation Metrics | GPT-3.5 | GPT-3.5 (fine-tuned) | GPT-4 | Davinci-003 | GAL-30B | MolT5-Large | LLama-2-7B | Llama2-13B-chat | BART-base | T5-base | T5-large | MolT5-base | MolT5-large | text-ada-001 (fine-tuned) | Ridge Regression | Neighbor (KNN) | ada embeddings | SolTranNet | SMILES-BERT | MolBERT | Regression Transformer | MolFormer |
| What can large language models do in chemistry? a comprehensive benchmark on eight tasks | Name Prediction ("smiles2formula") | PubChem | Accuracy | 0.052 ('Scaffold,k=20') | - | 0.086 ('Scaffold,k=20') | 0.006 ("Scaffold,k=20") | - | - | - | 0.01 ("Scaffold,k=20") | - | - | - | - | - | - | - | - | - | - | - | - | - | - |
| | Property Prediction | BBBP | F1 | 0.463 ('Scaffold,k=20') | - | 0.587 ('Scaffold,k=20') | 0.378 ('Scaffold,k=20') | 0.074 ('Scaffold,k=20') | - | - | 0.002 ('Scaffold,k=20') | - | - | - | - | - | - | - | - | - | - | - | - | - | - |
| | Property Prediction | HIV | F1 | 0.406 ('Scaffold,k=20') | - | 0.666 ('Scaffold,k=20') | 0.649 ('Scaffold,k=20') | 0.025 ('Scaffold,k=20') | - | - | 0.045 ('Scaffold,k=20') | - | - | - | - | - | - | - | - | - | - | - | - | - | - |
| | Property Prediction | BACE | F1 | 0.807 ('Scaffold,k=20') | - | 0.797 ('Scaffold,k=20') | 0.832 ('Scaffold,k=20') | 0.014 ('Scaffold,k=20') | - | - | 0.069 ('Scaffold,k=20') | - | - | - | - | - | - | - | - | - | - | - | - | - | - |
| | Property Prediction | Tox21 | F1 | 0.529 ('Scaffold,k=20') | - | 0.563 ('Scaffold,k=20') | 0.518 ('Scaffold,k=20') | 0.077 ('Scaffold,k=20') | - | - | 0.047 ('Scaffold,k=20') | - | - | - | - | - | - | - | - | - | - | - | - | - | - |
| | Property Prediction | ClinTox | F1 | 0.369 ('Scaffold,k=20') | - | 0.736 ('Scaffold,k=20') | 0.85 ('Scaffold,k=20') | 0.081 ('Scaffold,k=20') | - | - | 0.001 ('Scaffold,k=20') | - | - | - | - | - | - | - | - | - | - | - | - | - | - |
| | Yield Prediction | Buchwald-Hartwig | Accuracy | 0.585 ('random, k = 8') | - | 0.8 ('random, k = 8') | 0.467 ('random, k = 8') | 0 | - | - | 0.008 | - | - | - | - | - | - | - | - | - | - | - | - | - | - |
| | Yield Prediction | Suzuki-Miyaura | Accuracy | 0.542 ('random, k = 8') | - | 0.764 ('random, k = 8') | 0.341 ('random, k = 8') | 0.008 | - | - | 0.006 | - | - | - | - | - | - | - | - | - | - | - | - | - | - |
| | Reaction Prediction | USPTO-Mixed | Accuracy | 0.184 ('Scaffold, k=20') | - | 0.23 ('Scaffold, k=20') | 0.218 ('Scaffold, k=20') | 0.036 ('Scaffold, k=5') | - | - | 0.032 ('Scaffold, k=20') | - | - | - | - | - | - | - | - | - | - | - | - | - | - |
| | Reagents Selection | Suzuki-Miyaura | Top-1 Accuracy | 0.4 | - | 0.299 | 0.178 | 0.107 | - | - | 0.145 | - | - | - | - | - | - | - | - | - | - | - | - | - | - |
| | Retrosynthesis | USPTO-50k | Top-1Accuracy | 0.022 ('Scaffold, k=20') | - | 0.096 ('Scaffold, k=20') | 0.122 ('Scaffold, k=20') | 0.016 ('Scaffold, k=5') | - | - | 0 ('Scaffold, k=20') | - | - | - | - | - | - | - | - | - | - | - | - | - | - |
| | Text-Based Molecule Design | ChEBI-20 | BLEU | 0.479 ('Scaffold, k=10') | - | 0.816 ('Scaffold, k=10') | 0.741 ('Scaffold, k=10') | 0.004 | 0.601 | - | 0.626 ('Scaffold, k=10') | - | - | - | - | - | - | - | - | - | - | - | - | - | - |
| | Molecule Captioning | ChEBI-20 | BLEU-2 | 0.468 ('Scaffold,k=10') | - | 0.464 ('Scaffold,k=10') | 0.488 ('Scaffold,k=10') | 0.008 | 0.482 | - | 0.197 ('Scaffold,k=10') | - | - | - | - | - | - | - | - | - | - | - | - | - | - |
| Empowering molecule discovery for molecule-caption translation with large language models: A chatgpt perspective | Molecule Captioning | ChEBI-20 | BLEU-2 | 0.565 ('10-shot MolReGPT') | - | 0.607 ('10-shot MolReGPT') | - | - | - | 0.489 (2-shot MolReGPT) | - | - | 0.511 | 0.558 | 0.54 | 0.594 | - | - | - | - | - | - | - | - | - |
| | Text-Based Molecule Design | ChEBI-20 | BLEU | 0.79 ('10-shot MolReGPT') | - | 0.857 ('10-shot MolReGPT') | - | - | - | 0.693 (2-shot MolReGPT) | - | - | 0.762 | 0.854 | 0.769 | 0.854 | - | - | - | - | - | - | - | - | - |
| Bayesian optimization of catalysts with in-context learning | Property Prediction ("aqueous solubility") | ESOL | RMSE (the lower the better) | - | - | 0.773 ('topk') | 1.185 ('topk') | - | - | - | - | - | - | - | - | - | 1.558 ('topk') | - | 2.443 | 2.652 ('topk') | 2.99 | 0.47 | 0.531 | 0.73 | 0.278 |
| | Property Prediction ("reaction yield") | Nguyen et al. (2020) ACS Catal 10(2):921-932 | RMSE (the lower the better) | - | - | 2.683 ('topk') | 2.652 ('topk') | - | - | - | - | | - | - | - | - | 1.936 ('topk') | 2.114 ('topk') | 3.247 ('topk') | 4.173 ('topk') | - | - | - | - | - |
| Fine-tuning Large Language Models for Chemical Text Mining | Action Sequence extraction | Specified in Suppl Table but not accessible at time of writing | BLEU | 49.5 ('30-shots') | 84.8 (fine-tuned with 1060 samples) | 65.0 ('60-shots') | - | - | - | - | 81.6(fine-tuned with 1060 samples) | 74.4(fine-tuned with 1060 samples) | 84.1(fine-tuned with 1060 samples) | - | - | - | - | - | - | - | - | - | - | - | - |

Values are extracted from corresponding reference listed in the first column. In red are numbers that are no better than at least one kind of GPT. In paratheses are prompting strategies or additional settings detailed in the corresponding litetature.
